# Successful treatment of amoxicillin-induced linear IgA bullous dermatosis of childhood with rituximab: A case report and review of the literature

**DOI:** 10.1016/j.jdcr.2025.05.009

**Published:** 2025-06-09

**Authors:** Tiffany Liu, Emily Keyes, Alreem Al-Nabti, Nina Blank, Cynthia Magro

**Affiliations:** a Georgetown University School of Medicine, Washington, District of Columbia; b Israel Englander Department of Dermatology, Weill Cornell Medical College, New York, New York; c Department of Pathology and Laboratory Medicine, Weill Cornell Medicine, New York, New York

**Keywords:** amoxicillin-induced, anti-CD20 therapy, bullous dermatosis treatment, direct immunofluorescence, IgA autoantibodies, immunotherapy in dermatology, indirect immunofluorescence, linear IgA bullous dermatosis, pediatric autoimmune skin disorders, pediatric dermatology case report, refractory LABD treatment, rituximab therapy

## Introduction

Autoimmune vesiculobullous disorders in the pediatric age group encompass a spectrum of conditions characterized by blistering and erosions of the skin and mucous membranes. Among these diseases, linear IgA bullous dermatosis (LABD) is often idiopathic but may rarely be drug induced.^1^ Here, we present a case of amoxicillin-induced LABD in the youngest patient documented in the literature to date. This patient’s disease was highly refractory to therapy and was ultimately successfully treated with rituximab, highlighting a novel therapeutic approach for this rare condition.

## Case report

**Fig 1:**
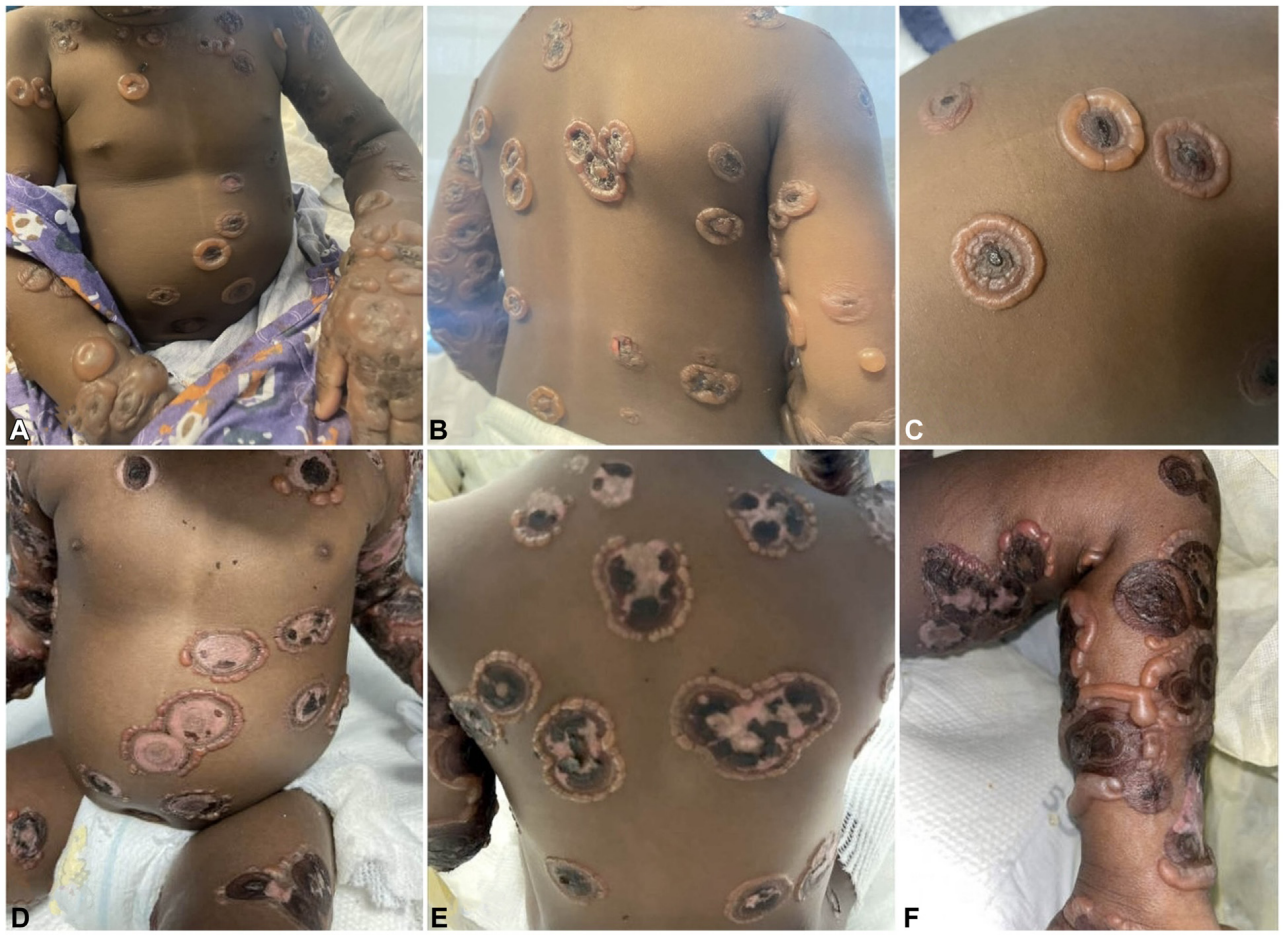
This 20-month-old female presented with numerous tense annular bullae with central crusting, many with characteristic “string of pearls” or “crown of jewels” pattern, on the trunk and extremities **(A-C)**. Despite treatment, the bullae continued to progress before the initiation of rituximab, developing into eroded hyper- and hypopigmented polycyclic and annular plaques with surrounding rim of tense vesicles again arranged in a “string of pearls” formation, on the trunk and extremities **(D-F)**.

A 20-month-old healthy female with recently diagnosed acute otitis media treated with amoxicillin presented with a new-onset blistering rash. Two days after completing a 10-day course of amoxicillin therapy for acute otitis media, she developed perioral blisters that spread to the trunk and extremities within the day. This was her first exposure to amoxicillin, and she had not received any other medications. On examination, she was found to have numerous tense vesicles and bullae arranged in a “string-of-pearls” formation forming annular plaques on her face, trunk, and extremities (Fig 1, *A*-*C*). She had no ocular or mucosal involvement.

**Fig 2:**
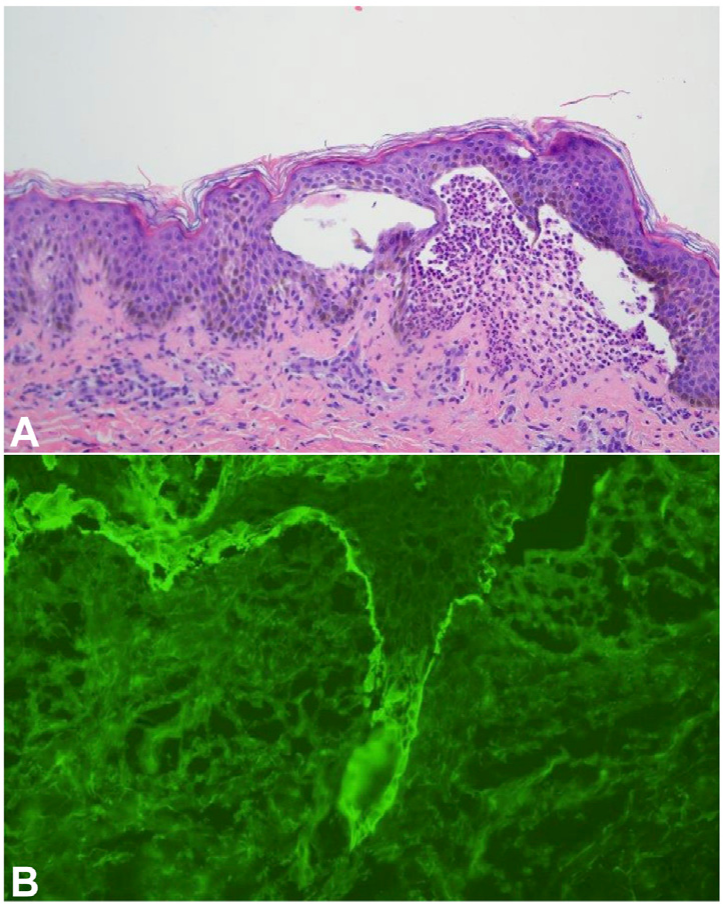
The biopsy shows a striking neutrophilic interface dermatitis with frank epidermal and dermal separation. Many neutrophils have accumulated in the blister cavity (**A**; Hematoxylin and eosin 200×). The biopsy shows an intense homogeneous linear staining pattern for IgA within the epidermal basement membrane zone (**B**; Fluorescent conjugated anti-IgA, 400×).

Punch biopsies were performed, and she was empirically started on intravenous (IV) corticosteroids. Light microscopy showed a neutrophilic interface dermatitis. There were coalescing dermal papillae microabscesses with resultant epidermal and dermal separation (Fig 2, *A*).

Direct immunofluorescence studies demonstrated intense and homogeneous linear IgA staining and discontinuous granular C5b-9 within the epidermal basement membrane zone, without significant immunoreactivity for IgG, IgM, C3, and C1q (Fig 2, *B*). Indirect immunofluorescence (IIF) studies on salt-split skin detected circulating IgA targeting the epidermal basement membrane zone. A diagnosis was made of LABD.

**Fig 3:**
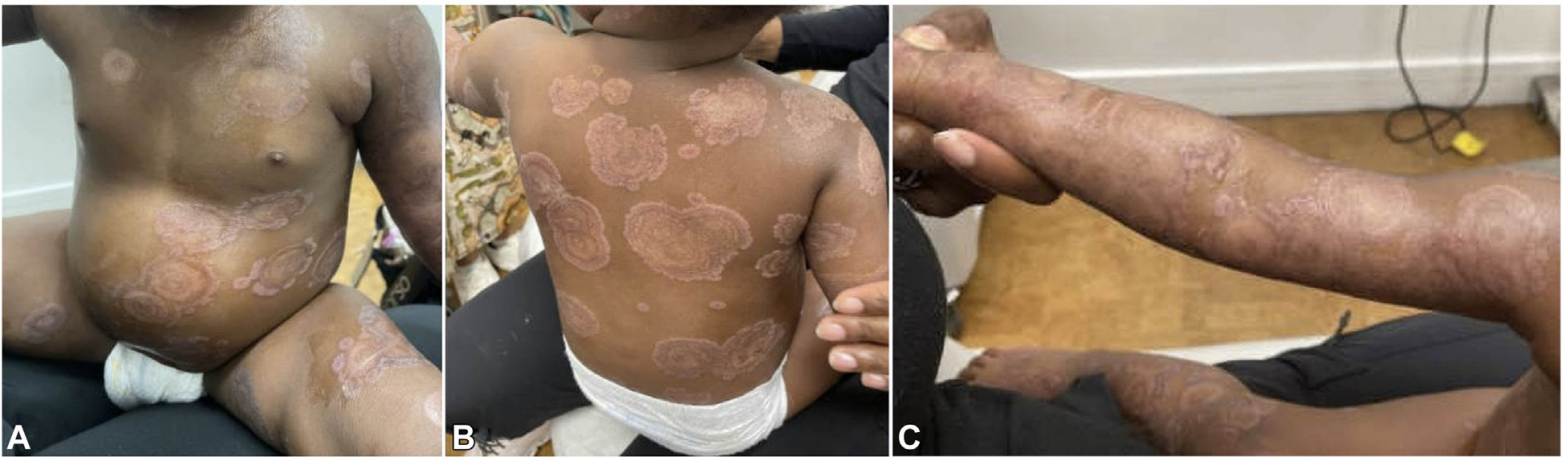
1.5 weeks after the first dose of rituximab, the patient showed significant improvement, with hypopigmented polycyclic and annular patches appearing at the sites of previous lesions on the trunk and extremities **(A-C)**.

Despite several days of IV corticosteroids 1-2 mg/kg/day, she continued to form new blisters (Fig 1, *D*-*F*). She was then started on dapsone 0.5 mg/kg/day after confirming normal G6PD levels, while continuing IV corticosteroids. However, additional blisters erupted including labial involvement. A decision was made to treat with intravenous immunoglobulin (IVIG) 2 g/kg and 3 days of pulsed IV corticosteroids 10-30 mg/kg/day. Concurrently, dapsone was uptitrated to 1 mg/kg/day. While this halted blister progression for a few days, she flared soon after decreasing the IV corticosteroid dose to 1-2 mg/kg/day. Given the refractory and progressive nature of her disease, rituximab therapy was started based on a positive treatment response to anti-CD20 therapy in treating both adult and pediatric LABD.^2^^,^^3^ She received one dose of rituximab 375 mg/m^2^, 3 days of pulsed IV corticosteroids 10 mg/kg/day, and continued dapsone. Immediately following first administration of rituximab, blister progression halted and existing blisters improved (Fig 3, *A*-*C*). She remained admitted for another week without new blister formation and discharged on oral steroids and dapsone. She developed 3-4 tiny new vesicles 10 days after the first dose of rituximab. A second dose was administered 2 weeks after the first, as per dosing protocol. Over the next month, she developed 1-2 tiny vesicles per week, which were controlled with topical steroids. Prednisone was carefully tapered off with the assistance of endocrinology and dapsone was continued. During the subsequent 8 months, she experienced 1-2 tiny new vesicles every few weeks, also controlled with topical steroids. Considering her previous significant flares despite high doses of dapsone and prednisone, along with her rapid, substantial, and sustained improvement after adding rituximab which allowed for significant prednisone tapering, her near-remission was attributed to rituximab. Despite a lasting clinical response, IgA antibodies remained detectable on IIF 3 months after rituximab administration.

## Discussion

This case highlights a refractory presentation of pediatric drug-associated LABD in a 20-month-old female. Though usually idiopathic, pediatric LABD has rarely been reported to be drug induced. While there are several documented cases of amoxicillin-induced LABD in adults, there are only 4 other reported pediatric cases.^4^, ^5^, ^6^, ^7^ Prior to this case, the youngest child reported was 3 years old.^4^ A summary of these previously reported cases is presented in Table I. In brief, the female to male ratio was 1:1 with patients ranging from 2.5 to 9 years of age. The rash appeared on average 12.3 days after starting amoxicillin and lasted an average of 3.5 months. The eruptions were self-limited and resolved with either drug cessation alone or in concert with additional therapy, encompassing dapsone, macrolides, cephalosporins, IVIG, oral steroids, IV steroids, and topical steroids.

**Table I:** Amoxicillin-induced linear IgA bullous dermatosis of childhood

Case	Age (y)/sex	Clinical presentation	Time from amoxicillin initiation to rash onset	Duration of rash	Successful treatment(s)	Failed treatments	DIF	Immunologic findings	Outcome
Current case Case report	1/F	Sudden onset of blistering vesicles and bullae on face, trunk, and extremities	12 d	1.5 wk	Withdrawal of amoxicillin and rituximab	Corticosteroids, dapsone, IVIG	Linear IgA and discontinuous granular C5b-9 along the BMZ	IIF: IgA (+)	Resolution of existing vesicles and bullae, with minimal new vesicles at 2 wk follow-up. Near-remission sustained over 3 mo.
Stamenkovic et al,^7^ 2020 Case report	9/M	Blistering vesicles and bullae on face, body, arms, hands, soles, perigenital, and perianal area	10 d	2 wk	Withdrawal of amoxicillin and macrolides, cephalosporins, systemic methylprednisolone, IVIG, dapsone	None	Linear IgA along the BMZ	NA	Resolution of skin changes was noticed without onset of new vesicles and bullae at 2 wk follow-up
Mori et al,^5^ 2020 Case report	2.5/F	Bullous, itchy rash with groups of new small blisters arising at the periphery of older bullae covering >50% of body surface	6 d	6 mo	Withdrawal of amoxicillin & systemic methylprednisolone, oral dapsone, oral prednisone	None	Linear IgA along the BMZ	NA	No relapses were observed after 2 mo, with complete remission at 6 mo
Garel et al,^4^ 2019 Case report	3/F	NA	NA	NA	Withdrawal of amoxicillin	NA	Linear IgA along the BMZ	IIF: IgA (+) ELISA: anti-collagen VII antibodies (+)	Complete remission
Ho et al,^6^ 2007 Case report	2/M	Recurrent acute-onset episodes of blistering eruptions on legs, neck, and right postauricular regions	3 wk	>4 mo	Withdrawal of amoxicillin-clavulanic acid & topical corticosteroid for recurrence	None	Linear IgA along the BMZ	IIF: IgA (−)	Minimal involvement at 4 mo follow-up, with an occasional blister, which resolved spontaneously within 2-3 d

*BMZ*, Basement membrane zone; *F*, female; *IVIG*, intravenous immunoglobulin; *M*, male; *mo*, months; *NA*, not available; *wk*, weeks.

There are currently no Food and Drug Administration approved medications for the treatment of LABD, but dapsone and corticosteroids are first-line treatments.^8^ Unlike previously reported pediatric amoxicillin-induced LABD cases, our case was refractory to high dose corticosteroids, dapsone, and IVIG, ultimately requiring treatment with rituximab, which was successful. This case represents both the youngest reported case as well as the only case of drug-induced LABD treated with rituximab. This treatment approach, while novel, draws on experience from other cases of refractory LABD managed with rituximab, though those cases were idiopathic rather than drug induced. Agarwal et al described a 5-year-old girl with idiopathic LABD successfully treated with rituximab after failing multiple therapies, including corticosteroids, dapsone, azathioprine, and IVIG.^3^ Mitra et al reported a 2-year-old boy with a similar presentation also successfully treated with rituximab (Table I).^2^

While clinical remission following rituximab was notable, IIF studies indicated persistent IgA antibodies 3 months after administration, which contrasts with the clinical resolution of disease. The pathogenesis of drug-induced LABD is thought to involve the drug acting as a hapten, modifying the antigenic profile of the BMZ and inciting an immune response, resulting in IgA deposition.^1^ Rituximab targets CD20 to deplete B cells and reduce autoantibody production.^9^ Since mature plasma cells lack CD20, they are unaffected by rituximab.^9^ As a result, autoantibodies can persist in the absence of active disease. A previous study found no association between autoimmune disease response to rituximab and hypogammaglobulinemia.^10^ Discrepancy between rituximab-mediated B cell depletion and persistent autoantibody levels, along with failure of IVIG treatment, suggests potential alternative roles for B cells in LABD pathogenesis beyond autoantibody production.

In summary, we have presented the youngest reported case of amoxicillin-induced LABD and first drug-induced LABD treated with rituximab. Further research is needed to understand rituximab's mechanism, safety, and efficacy in treating refractory pediatric LABD.

## Conflicts of interest

None disclosed.
